# Ringing phenomenon based whispering-gallery-mode sensing

**DOI:** 10.1038/srep19597

**Published:** 2016-01-22

**Authors:** Ming-Yong Ye, Mei-Xia Shen, Xiu-Min Lin

**Affiliations:** 1 Fujian Provincial Key Laboratory of Quantum Manipulation and New Energy Materials, College of Physics and Energy, Fujian Normal University, Fuzhou 350007, China

## Abstract

Highly sensitive sensing is one of the most important applications of whispering-gallery-mode (WGM) microresonators, which is usually accomplished through a tunable continuous-wave laser sweeping over a whispering-gallery mode with the help of a fiber taper in a relative slow speed. It is known that if a tunable continuous-wave laser sweeps over a high quality whispering-gallery mode in a fast speed, a ringing phenomenon will be observed. The ringing phenomenon in WGM microresonators is mainly used to measure the Q factors and mode-coupling strengths. Here we experimentally demonstrate that the WGM sensing can be achieved based on the ringing phenomenon. This kind of sensing is accomplished in a much shorter time and is immune to the noise caused by the laser wavelength drift.

Whispering-gallery-mode (WGM) microresonators with different geometry have been intensively investigated in recent years^1^,^2^,^3^,^4^,^5^,^6^,^7^,^8^,^9^,^10^. These optical microresonators can support whispering-gallery modes with high quality (Q) factors and small mode volumes. They have been found wide range of applications such as low threshold lasing^11^,^12^,^13^, highly sensitive sensing^14^,^15^,^16^,^17^,^18^,^19^,^20^,^21^, nonlinear optics^22^,^23^,^24^, cavity quantum electrodynamics^25^, optomechanics^26^,^27^, and simulation of fundamental physics^28^,^29^,^30^. Among the applications of WGM microresonators, highly sensitive sensing attracts special attentions. In the ordinary WGM sensing, a tunable continuous-wave laser is used to sweep over a whispering-gallery mode in a relative slow speed and usually the transmission spectrum will have a Lorentz shape. If the environment of the WGM microresonator changes, e.g., a biomolecule or nanoparticle gets into the evanescent field of the microresonator, the Lorentz-shape transmission spectrum will have some variation, such as the resonance wavelength shift^17^, the mode broadening^15^,^19^ and splitting^16^. The environmental change can then be sensed by measuring the variation of the transmission spectrum.

It is known that when a tunable continuous-wave laser sweeps over a high Q whispering-gallery mode with the help of a fiber taper in a fast speed, the transmission spectrum will not be a Lorentz shape but some oscillation can be observed, which is called the ringing phenomenon^31^,^32^,^33^,^34^. The ringing phenomenon is first reported in a two-mirror cavity, and it now provides a highly sensitive way to measure the optical absorption of the gas sample between the mirrors^35^. It has also been discussed in fiber loop resonators to sensor the environmental change^36^. In WGM microresonators, the ringing phenomenon is mainly used to measure the characteristics of the modes such as the Q factors and mode-coupling strengths^32^,^37^. Here we experimentally demonstrate that the ringing phenomenon can be used to sense the environmental change of the WGM microresonator. In the experiment, the environmental change leads to some variation in the observed ringing phenomenon. Conversely, from the variation of the ringing phenomenon the environmental change can be sensed.

## Results

### Theory and sensing mechanism

**Figure 1:**
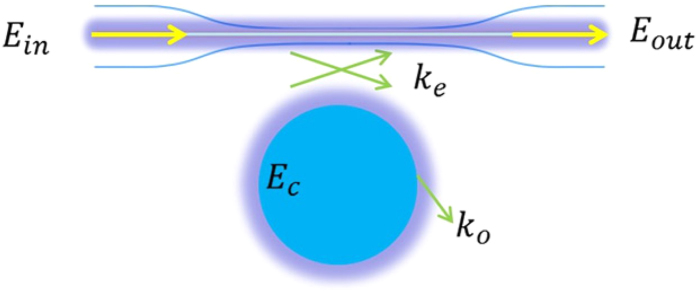
Sketch view of a microsphere and fiber taper system. Light from a tunable continuous-wave laser is coupled into and out of a silica microsphere through a fiber taper.

Figure 1 presents a sketch of the experimental configuration, where a fiber taper^38^ is used to couple laser into and out of a silica microsphere^25^. The theory involved is as follows. Suppose the light field in the microsphere is denoted by *E*_*c*_(*t*) and it is normalized so that |*E*_*c*_(*t*)|^2^ represents the energy stored in the microsphere. The input field and the output field in the fiber taper is denoted by *E*_*in*_(*t*) and *E*_*out*_(*t*), respectively. They are normalized so that |*E*_*in*_(*t*)|^2^ represents the input power and |*E*_*out*_(*t*)|^2^ represents the output power. The input is controlled by a tunable continuous-wave laser, and the output is linked to the input by the following equations^33^,^34^


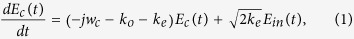



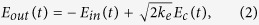


where *w*_*c*_ is the resonance angular frequency of the whispering-gallery mode, which can be regarded as a constant by assuming that the laser intensity is weak. The total loss rate of the mode is *k*_*o*_ + *k*_*e*_, where *k*_*o*_ is the intrinsic loss rate and *k*_*e*_ is the loss rate associated with the fiber taper coupling. The normalized transmission of the fiber taper in the time domain is


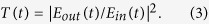


In the experiment, the angular frequency of the laser sweeps (decreases) linearly in time and the input field can be modeled as


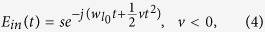


where *s* represents the amplitude of the input field. By differentiating the total phase of *E*_*in*_(*t*) with time *t*, it can be found that the instantaneous angular frequency of the input field is 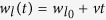. Suppose at the initial time *t* = 0 there is *E*_*c*_ = 0 and 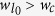, the laser will sweep over the resonance angular frequency *w*_*c*_ of the whispering-gallery mode in a constant speed *v*. How *T*(*t*) (*t* > 0) changes with time *t* can be obtained through a numerical solution of Eq. (1). The function shape of *T*(*t*) will depend on *k*_*o*_, *k*_*e*_ and the laser sweeping speed *v*.

**Figure 2:**
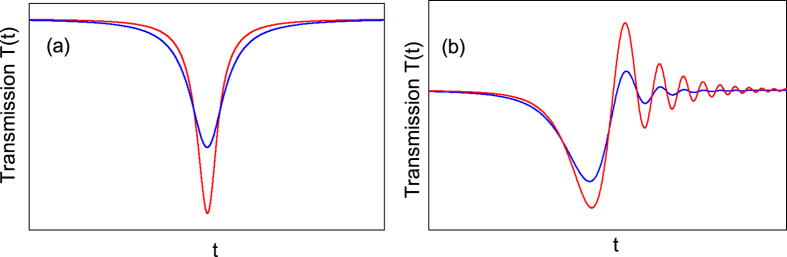
Sketch demonstration of sensing mechanism in the undercoupling. The laser sweeping speed is slow in (**a**) and fast in (**b**). The blue curves have larger intrinsic loss rates than the red ones.

The laser sweeping speed *v* has a significant role on the shape of the transmission *T*(*t*). Define *v*_0_ = 4(*k*_*o*_ + *k*_*e*_)^2^, which is corresponding to the speed that sweeps over the mode width 2(*k*_*o*_ + *k*_*e*_) within a cavity lifetime 1/2(*k*_*o*_ + *k*_*e*_). Generally speaking^34^, if the magnitude of the laser sweeping speed *v* is much smaller than *v*_0_, the laser sweeping speed can be regarded as slow; if the magnitude of the laser sweeping speed *v* is comparable or larger than *v*_0_, the laser sweeping speed can be regarded as fast. Figure 2 gives some sketches of the transmission *T*(*t*) in the undercoupling (*k*_*o*_ > *k*_*e*_). In Fig. 2(a) the laser sweeping speed is slow and the transmission *T*(*t*) has a Lorentz shape^34^. While in Fig. 2(b), the laser sweeping speed is fast and some oscillation is observed^34^, which is called the ringing phenomenon. The ringing phenomenon results from the interference between the directly transmitted light and the light leaked from the microsphere. It can be found from Fig. 2(a) that as only the intrinsic loss rate *k*_*o*_ of the microsphere increases the Lorentz-shape transmission will broaden, which is the sensing mechanism studied before^15^,^19^. It can be found from Fig. 2(b) that as only the intrinsic loss rate *k*_*o*_ increases, the oscillation will become less obvious. Since the environmental change may lead to some change of the intrinsic loss rate, it may lead to some variation in the observed ringing phenomenon. Therefore it is possible to sense the environmental change from the ringing phenomenon. This is the sensing mechanism that will be explored. We note that when the laser intensity is relatively large, the resonance angular frequency *w*_*c*_ in Eq. (1) will not be a constant and a theory with thermal effect should be used^31^.

### Experimental setup

**Figure 3:**
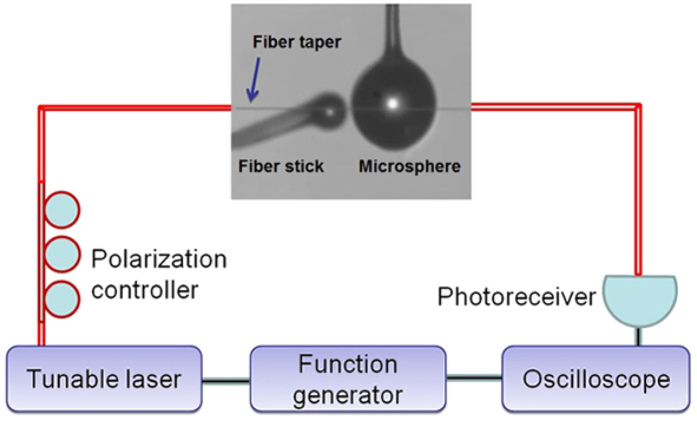
Experimental setup. The fiber stick is placed far below the fiber taper so that there is no coupling between them.

The main experimental setup is shown in Fig. 3. A tunable continuous-wave laser in the 1550 nm band with a linewidth smaller than 200 kHz is used (TLB-6728, Newport). The sweeping of the laser is controlled by a triangle-wave signal from a function generator. The triangle-wave signal is also monitored on a digital oscilloscope. A fiber taper with a diameter about 2 *μ*m is used to couple laser into and out of a silica microsphere. The polarization controller is used to tune the polarization of the light in the fiber taper. The air gap between the fiber taper and the microsphere is controlled by a translation stage with a resolution of 20 nm that holds the microsphere (MAX312D, Thorlabs). The transmitted light from the fiber taper is measured by a 125 MHz low-noise photoreceiver (1811-FC, Newport), whose output signal is sent to the digital oscilloscope to obtain the transmission *T*(*t*). A fiber stick is used to control the environmental change of the microsphere, which is hold by another translation stage with a resolution of 20 nm (MAX312D, Thorlabs). The fiber taper is placed above the microsphere while the fiber stick is kept far below the fiber taper so that there is no coupling between the fiber taper and the stick. The reflection light from the microsphere via the fiber taper is also measured by a 125 MHz low-noise photoreceiver (not shown in Fig. 3), which is used to check whether there is mode coupling between the clockwise (CW) mode and counterclockwise (CCW) mode in the microsphere (no mode coupling is observed for the modes studied below)^16^. The fabrication of the fiber taper, the microsphere and the fiber stick is described in the section of Methods.

### Measurements

**Figure 4:**
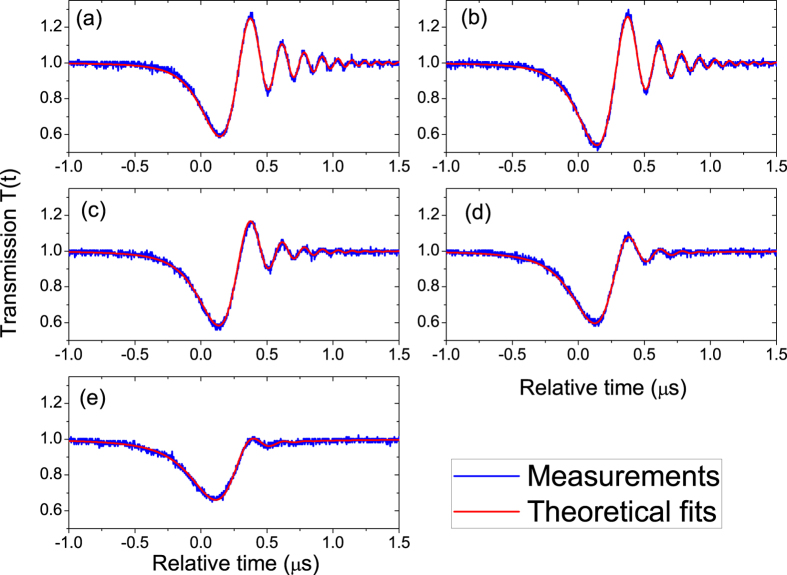
Experimental transmissions and theoretical fits. From (**a**–**e**) the fiber stick is placed closer and closer to the microsphere but the laser sweeping speed is unchanged.

A silica microsphere with the diameter about 115 *μ*m was studied. A tunable continuous-wave laser swept over a whispering-gallery mode with the resonance wavelength about 1534 nm in a fast speed. The laser intensity was kept low so that the thermal effect in the microsphere could be ignored. In the experiment, the fiber stick was placed on several positions and the transmissions were recorded respectively, where the largest distance between the fiber stick and the microsphere is about one wavelength. Figure 4 shows the experimental transmissions and theoretical fits. From Fig. 4(a–e) the fiber stick was placed closer and closer to the microsphere but the laser sweeping speed was unchanged. It is obvious that the ringing phenomenon is affected by the the position of the fiber stick. It can be found that the theoretical fits agree well with the experimental measurements. Therefore the ringing phenomenon can be used to measure the loss rates *k*_*o*_ and *k*_*e*_ of the microsphere through theoretical fits^33^.

The fitting parameters are shown in Table 1. Note that there is always *k*_*o*_ > *k*_*e*_, therefore the experiment is done in the undercoupling. It can be found in Table 1 that both the intrinsic loss rate *k*_*o*_ and the loss rate *k*_*e*_ increase from Fig. 4(a–e). Note that the loss rate *k*_*e*_ is associated with the fiber taper coupling. That *k*_*e*_ has some little changes means that the gap between the fiber taper and the microsphere is not very stable in the experiment. It is not easy to keep the gap stable in the experiment, but we will show that the variation of the gap has little effect on the intrinsic loss rate *k*_*o*_ in the undercoupling. Therefore we conclude that the increasing of *k*_*o*_ in our experiment is due to the fact that the fiber stick is moved closer to the microsphere that increases the light scattering and absorption. It means that the environmental change of the microsphere is sensed through the variation of the observed ringing phenomenon. We note that there are other specific features of the ringing phenomenon that may be used to sense the environmental change. For example, as shown in Fig. 4 the decay rate of the ringing peak amplitude and the ringing peak-to-peak amplitude (the maximum minus the minimum) are different with different positions of the fiber stick (see Table 1). Therefore, sensing may be achieved by observation of the variation of these values. However, the variation of these values not only depends on the change of *k*_*o*_ caused by the fiber stick but also depends on the change of *k*_*e*_ caused by the fiber taper. So the vibration of the fiber taper can cause some noise to these sensors. However, in the sensing mechanism we just demonstrated the value of *k*_*o*_ can be obtained by fitting the experimental data, whose change is only caused by the fiber stick.

**Figure 5:**
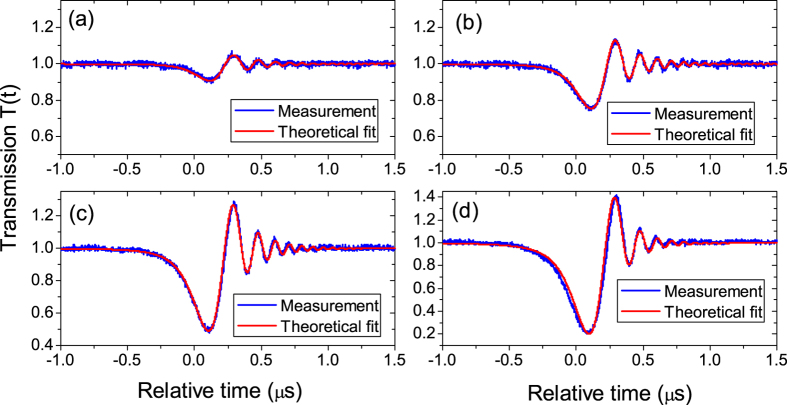
Demonstration that the gap between the fiber taper and the microsphere has little effect on the intrinsic loss rate of the microsphere.

To show that the variation of the gap between the fiber taper and the microsphere has little effect on the intrinsic loss rate *k*_*o*_ of the microsphere, a new experiment was done on a silica microsphere with a diameter about 52 *μ*m. In the new experiment, a high Q mode with the resonance wavelength about 1541 nm was swept over in a fast speed, where there was no the fiber stick. We only changed the gap between the fiber taper and the microsphere while keeping the fast laser sweeping speed unchanged. The experimental result with several gap distance is shown in Fig. 5. From Fig. 5(a–d), the microsphere was placed closer and closer to the fiber taper, where the largest distance between the microsphere and the fiber taper is several micrometers. As the gap becomes smaller, the loss rate *k*_*e*_ will become larger. The fitting parameters are shown in Table 2. It can be seen that when *k*_*e*_ changes from much smaller than *k*_*o*_ (Fig. 5(a)) to near *k*_*o*_ (Fig. 5(d)) by placing the microsphere closer to the fiber taper, the theoretical fits all agree well with the experimental measurements, which demonstrates that the gap between the fiber taper and the microsphere almost has no effect on the intrinsic loss rate *k*_*o*_ of the microsphere in the undercoupling (*k*_*o*_ > *k*_*e*_).

## Discussion

The ringing phenomenon can be observed only when the laser sweeping speed is fast. It has been mentioned that if the magnitude of the laser sweeping speed *v* is comparable or larger than *v*_0_ = 4(*k*_*o*_ + *k*_*e*_)^2^, the laser sweeping speed can be regarded as fast. Note that the quality factor of the mode is *Q* = *w*_*c*_/2(*k*_*o*_ + *k*_*e*_), it can be found that *v*_0_ = (*w*_*c*_/*Q*)^2^. Suppose *Q* = 1.0 * 10^8^ and the mode resonant wavelength is 1500 nm (*w*_*c*_/2*π* = 2 * 10^8^ MHz), there is *v*_0_ ≈ 2*π* * 25 MHz/*μ*s or 188 nm/s. If the quality factor Q is reduced from 1.0 * 10^8^ to 1.0 * 10^7^, *v*_0_ will be increased from 188 nm/s to 18800 nm/s that is a speed not easy to be achieved in experiment. In our sensing experiment, the laser sweeping speed is *v* = −2*π* * 8.6 MHz/*μ*s. If we use the fitting parameters from Fig. 4(a) where the ringing phenomenon is clear, there is *v*_0_ ≈ 2*π* * 7.3 MHz/*μ*s that is smaller than the magnitude of the laser sweeping speed in the experiment; If we use the fitting parameters from Fig. 4(e) where the ringing phenomenon is not clear, there is *v*_0_ ≈ 2*π* * 45 MHz/*μ*s that is larger than the magnitude of the laser sweeping speed in the experiment. Both the Q factor of the mode and the laser sweeping speed have important roles in the observation of the ringing phenomenon.

The fiber stick in the experiment is a relatively big object. It is an interesting question that whether the demonstrated sensing mechanism can be used to detect smaller analytes such as nanoparticles. Detection of 70 nm-radius polystyrene (PS) particles has been demonstrated by monitoring mode broadening in deformed microtoroids^15^, where the minimal mode broadening caused by an individual nanoparticle is 2*π* * 5.3 MHz (angular frequency). Though there are some difference between microspheres and microtoroids, based on the above data we estimate that the increase of *k*_*o*_/2*π* of a microsphere caused by an individual 70 nm-radius PS particle has the order of MHz, which is larger than the demonstrated increase of *k*_*o*_/2*π* as shown in Table 1. Therefore the ringing phenomenon has the potential to detect smaller analytes, but questions such as mode coupling may be needed to be considered.

In summary we have provided an experimental demonstration that the ringing phenomenon of a WGM microresonator can be used to sense the environmental change. A faster laser sweeping speed is used in the demonstration, so the sensing is accomplished in a shorter time than those based on the resonance wavelength shift, broadening and splitting. Therefore it can be used to sense faster processes as recently discussed in cavity ring-up spectroscopy^39^. In addition the sensing does not depend on the resonance wavelength shift, so it is immune to the noise caused by the slow laser wavelength drift. During the preparation of the manuscript, a theoretical WGM sensing method is proposed, which shows that the sensor based on the ringing phenomenon not only has the advantages such as high sensitivity and high temporal resolution but also has the potential to sense the speed of a moving nanoparticle^40^.

## Methods

The silica microspheres were fabricated by first preparing a thin fiber tip and then melting the tip through a CO_2_ laser^25^. The fiber stick was fabricated by using a method similar to the fabrication of the microspheres. The prepared microsphere and fiber stick were hold by fiber chucks, which could be fixed on translation stages. The fiber tapers were made by heating a single-mode fiber using a hydrogen flame while stretching the fiber from two sides by a motorized translational stage^38^. The prepared fiber taper was then fixed on a U-shape frame by some UV glue, so we could move the U-shape frame to move the fiber taper.

## Additional Information

**How to cite this article**: Ye, M.-Y. *et al.* Ringing phenomenon based whispering-gallery-mode sensing. *Sci. Rep.* **6**, 19597; doi: 10.1038/srep19597 (2016).

## Figures and Tables

**Table 1:** Fitting parameters for fits in Fig. 4 and the ringing peak-to-peak amplitude.

	a	b	c	d	e
*k*_*o*_/2*π* (MHz)	0.44	0.48	0.63	0.88	1.20
*k*_*e*_/2*π* (MHz)	0.10	0.12	0.12	0.14	0.14
peak-to-peak amplitude	0.66	0.74	0.59	0.49	0.34

All fits use the same laser sweeping speed *v* = −2*π* * 8.6 MHz/*μ*s.

**Table 2:** Fitting parameters for fits in Fig. 5.

	a	b	c	d
*k*_*o*_/2*π* (MHz)	0.66	0.66	0.66	0.66
*k*_*e*_/2*π* (MHz)	0.026	0.075	0.183	0.380

All fits use the same laser sweeping speed *v* = −2*π* * 14.3 MHz/*μ*s.
